# The [*PSI* ^+^] Prion Exists as a Dynamic Cloud of Variants

**DOI:** 10.1371/journal.pgen.1003257

**Published:** 2013-01-31

**Authors:** David A. Bateman, Reed B. Wickner

**Affiliations:** Laboratory of Biochemistry and Genetics, National Institute of Diabetes and Digestive and Kidney Diseases, National Institutes of Health, Bethesda, Maryland, United States of America; University of Nevada, Reno, United States of America

## Abstract

[*PSI* ^+^] is an amyloid-based prion of Sup35p, a subunit of the translation termination factor. Prion “strains” or “variants” are amyloids with different conformations of a single protein sequence, conferring different phenotypes, but each relatively faithfully propagated. Wild *Saccharomyces cerevisiae* isolates have *SUP35* alleles that fall into three groups, called reference, Δ19, and E9, with limited transmissibility of [*PSI* ^+^] between cells expressing these different polymorphs. Here we show that prion transmission pattern between different Sup35 polymorphs is prion variant-dependent. Passage of one prion variant from one Sup35 polymorph to another need not change the prion variant. Surprisingly, simple mitotic growth of a [*PSI* ^+^] strain results in a spectrum of variant transmission properties among the progeny clones. Even cells that have grown for >150 generations continue to vary in transmission properties, suggesting that simple variant segregation is insufficient to explain the results. Rather, there appears to be continuous generation of a cloud of prion variants, with one or another becoming stochastically dominant, only to be succeeded by a different mixture. We find that among the rare wild isolates containing [*PSI* ^+^], all indistinguishably “weak” [*PSI* ^+^], are several different variants based on their transmission efficiencies to other Sup35 alleles. Most show some limitation of transmission, indicating that the evolved wild Sup35 alleles are effective in limiting the spread of [*PSI* ^+^]. Notably, a “strong [*PSI* ^+^]” can have any of several different transmission efficiency patterns, showing that “strong” versus “weak” is insufficient to indicate prion variant uniformity.

## Author Summary

The [*PSI* ^+^] prion (infectious protein) of yeast is a self-propagating amyloid (filamentous protein polymer) of the Sup35 protein, a subunit of the translation termination factor. A single protein can form many biologically distinct prions, called prion variants. Wild yeast strains have three groups of Sup35 sequences (polymorphs), which partially block transmission of the [*PSI* ^+^] prion from cell to cell. We find that [*PSI* ^+^] variants (including the rare [*PSI* ^+^] from wild yeasts) show different transmission patterns from one Sup35 sequence to another. Moreover, we find segregation of different prion variants on mitotic growth and evidence for generation of new variants with growth under non-selective conditions. This data supports the “prion cloud” model, that prions are not uniform structures but have an array of related self-propagating amyloid structures.

## Introduction

Prions in yeast are a new form of gene, composed of proteins instead of nucleic acids [1]. As such, their inheritance, mutation and segregation are not expected to follow the same rules as the majority DNA/RNA genes. The [*PSI* ^+^] prion was first recognized as a non-chromosomal genetic element enhancing the read-thru of the premature termination codon in *ade2-1* [2]. Its unusual genetic properties led to its identification as a prion of Sup35p [1], a subunit of the translation termination factor [3], [4], specifically an amyloid form (β-sheet-rich filamentous polymer of protein subunits) of the normally soluble Sup35p [5]–[9]. In the amyloid form, the protein is largely inactive, resulting in increased read-through of termination codons. Yeast prions are important models for mammalian prion diseases, and for amyloid diseases in general.

Sup35p consists of C, an essential C-terminal domain (residues 254–685), responsible for the translation termination function [3], [4], [10]; N, an N-terminal domain necessary for prion propagation (residues 1–123) [10] that normally functions in the general mRNA turnover process [11]–[15] and functionally interacts with Sla1p [16]; and M (residues 124–253), a middle charged region that is also implicated in prion propagation [17]–[20]. In the infectious amyloid form, the N domain, and probably part of the M domain, is in an in-register parallel β-sheet form, with folds in the sheet along the long axis of the filament [21], [22].

Prions can often be transmitted between species, as was first recognized by infectivity of sheep scrapie brain extracts for goats [23]. However, cross-species transmission is inefficient (or completely blocked) as a result of sequence differences between the donor and recipient prion proteins [24]. This phenomenon is called the species barrier, and has also been observed in yeast prions [19], [25]–[31]. Wild isolates of *S. cerevisiae* also show considerable sequence variation in Sup35p sequence [20], [32], and these sequence differences produce barriers to transmission of [*PSI* ^+^] [20], presumably evolved to protect cells from the detrimental, even lethal, effects of this prion [33], [34].

A single prion protein can propagate any of a number of prion variants (called ‘prion strains’ in mammals), with biological differences due to different self-propagating conformations of the amyloid [9], [35], [36]. Although there is evidence for conformational differences between prion variants, the nature of those differences is not yet known. In yeast, prion variants differ in intensity of the prion phenotype, stability of prion propagation, interactions with other prions, response of the prion to overproduction or deficiency of various chaperones, and ability to cross species barriers [30], [31], [37]–[41]. Different variants arise during prion generation as a result of some stochastic events occurring in the initial formation of the prion amyloid. Generally, prion variant properties are rather stable, even during propagation in a species different from that in which the prion arose (e.g. [42]).

In a previous report, we demonstrated transmission barriers between Sup35 alleles from wild strains of *S. cerevisiae*, an ‘intraspecies barrier’. These intraspecies barriers are of particular interest since they must operate in nature, when S. cerevisiae strains mate among themselves. Interspecies matings are less efficient than intraspecies matings (e.g., [43]), and diploids formed produce almost no viable meiotic spores [44], [45]. In most cases, the intraspecies barriers were incomplete, with occasional transmission between strains with different Sup35 sequences. Were the prions transmitted the same variant as the original, or were they prion ‘mutants’, heritably changed in their properties? Under selective conditions, prion variant properties may change, a phenomenon first demonstrated in mice [46] and also known in yeast [30], [47]. Selection in the presence of a different prion protein sequence, or a drug interacting with amyloid could induce a new prion by inaccurate cross-seeding, and reflect generation of a new prion, rather than propagation of one of several sub-variants already present. Here, we examined variation in prion properties under non-selective conditions, finding evidence for the existence of a ‘cloud’ of variants with stochastic fluctuation.

## Results

### Prion variant-specificity of intraspecies transmission barriers

Wild *SUP35* alleles fall into three groups: the ‘reference’ sequence is essentially that of laboratory strains; Δ19 has a 19 residue (66–84) deletion in the prion domain; E9 is representative of a group with N109S and several polymorphisms in the M domain [20]. Three independent prion variants of the E9 Sup35p (E9A, E9F, E9G) were selected in strain 4828 (Table S1). We tested the transmission of these variants by cytoduction to strain 4830 expressing E9 itself, Δ19 or reference Sup35. None of these variants were transmitted well into the strain containing the Δ19 Sup35 polymorph. However, two variants (A, G) propagated very poorly with reference Sup35 sequence, while the other variant (F) was able to efficiently transmit the prion to the reference sequence (Table 1, p<10^−10^). This indicates that intraspecies transmission barriers are variant-specific.

**Table 1:** Variable transmission of [*PSI* ^+^E9]E9 isolates A, F, and G to polymorphs Sup35ref, Sup35E9, and Sup35Δ19 shows that they are distinct prion variants.

Donor	Recipient allele	Ade+ cytoductant	Total cytoductants	% Ade+
[*PSI^+^*E9A]	E9	75	80	94
	Δ19	5	85	6
	Reference	10	100	10*
[*PSI^+^*E9F]	E9	50	70	71
	Δ19	0	72	0
	Reference	60	69	87*
[*PSI^+^*E9G]	E9	**75**	88	85
	Δ19	**15**	82	18
	Reference	**12**	80	15*

Three prion isolates (A, F, G) in strain 4828 expressing the E9 polymorph of Sup35 were used as cytoduction donors to strain 4830 expressing the different polymorphs. **Bold** figures show which cytoductants were used as donors in Table 2. The proportions of transmission by variant E9A and E9G to the reference sequence differs from the proportion observed for variant E9F (*) with p<10^−10^, calculated as described in Methods.

Two of the few E9G→Δ19 cytoductants that were [*PSI* ^+^] (Table 1) were tested for transmission to strains with different Sup35 polymorphs (Table 2). Each had lost the transmission specificity and were now able to transmit the prion more efficiently into all sequences (Table 2, p<10^−10^), unlike two [*PSI* ^+^] isolates initially selected in cells expressing Δ19, which propagated poorly to E9 or reference [20] (p values between 10^−10^ and .002). This again shows the prion variant specificity of transmission barriers. Note that these two E9G→Δ19 cytoductants differ in that [*PSI* ^+^E9G]Δ19A was white (a strong [PSI+]) while [PSI+E9G]Δ19B was pink (a weak [PSI+]). This indicates that either the original E9G was a mixture of two prions or that new prion variants were selected by the difficulty of transmission into Δ19.

**Table 2:** Propagation characteristics of [*PSI* ^+^E9G] carried by different Sup35 polymorphs.

Donor	Recipient allele	Ade+ cytoductant	Total cytoductants	% Ade+
[*PSI^+^*E9G]Δ19A	E9	27	55	49
white	Δ19	70	78	90
	Reference	75	75	100
[*PSI^+^*E9G]Δ19B	E9	30	48	63
pink	Δ19	56	70	80
	Reference	70	70	100
[*PSI* ^+^E9G]Ref	E9	38	55	69
	Δ19	18	87	21
	Reference	70	70	100
[*PSI^+^*E9G]E9	E9	55	60	92
	Δ19	5	91	5
	Reference	18	55	33

[*PSI* ^+^E9G] cytoductants from Table 1 in strain 4830 were transmitted from the three Sup35 polymorphs to the three polymorphs in 4828. “[*PSI* ^+^E9G]Δ19A” means [*PSI* ^+^] variant G isolated originally in a cell expressing the E9 polymorph of Sup35p, but now propagating in a cell expressing Sup35Δ19, and cytoductants ‘A’. The donors here are cytoductants from Table 1. The p values for specific comparisons are given in the text.

An E9G→ref cytoductant from Table 1, similarly analyzed, showed ready propagation into reference (100%, p<10^−10^) and the original E9 sequence from which the prion originated (69%), but only poor transmission to the Δ19 sequence (Table 2). This result differs from a [*PSI* ^+^ref]ref (originating and propagating in the ref sequence) which propagates poorly into E9 (19%, p<10^−8^) [20], again showing prion variant dependence of prion transmission. As expected the E9G prion transmitted to another yeast strain with the E9 Sup35 had similar propagation characteristics to the original [*PSI* ^+^E9G] (compare Table 1 and Table 2).

The [*PSI* ^+^ref]ref in strain 779-6A was transmitted to cells with the other Sup35p polymorphs and, as expected, transmission was limited (Table 3). When [*PSI* ^+^] cytoductants were examined for stability on extensive further mitotic growth, we found that the [*PSI* ^+^ref]ref cytoductants were fully stable, while the [*PSI* ^+^ref]Δ19 were significantly less stable and [*PSI* ^+^ref]E9 cytoductants even less so. Nonetheless, stability was sufficient that [*PSI* ^+^ref]Δ19→Δ19 and [*PSI* ^+^ref]E9→E9 cytoductions showed >90% transmission (Table 3).

**Table 3:** Transmission of 779-6A's [*PSI* ^+^ref] carried by other Sup35 polymorphs.

Donor	Recipient allele	Ade+ cytoductant	Total cytoductants	% Ade+
[*PSI^+^* 779-6A]	Reference	**118**	120	98
	Δ19	**13**	122	11
	E9	**19**	111	17
**[** ***PSI^+^*** ** 779-6A]Δ19**	779-6A cured	212	226	94
	Reference	**50**	60	83
	Δ19	98	108	91
	E9	4	90	4
**[** ***PSI^+^*** ** 779-6A]E9**	779-6A cured	204	204	100
	Reference	**89**	94	95
	Δ19	5	80	6
	E9	104	113	92
**[** ***PSI^+^*** ** 779-6A]Ref**	779-6A cured	222	222	100
	Reference	**67**	67	100
	Δ19	5	72	7
	E9	13	78	17

The bold indicates cytoductants used as donors in a subsequent cytoduction.

The variant-dependence of transmissibility was again evident in cytoduction of [*PSI* ^+^ref]ref in strain 779-6A [48] to cells with the other Sup35p polymorphs (Table 3). This variant originated in the reference sequence, but when transferred to Sup35Δ19, is then transferred well to either the reference or the Δ19 Sup35s, but very poorly to E9 (Table 3). In contrast, either of two E9-originating prions in a Δ19 host ([*PSI* ^+^E9G]Δ19), transfer well to all polymorphs (Table 2, p<10^−10^). The [*PSI* ^+^ref]E9 transfers well to both reference and E9 sequences (Table 3), like [*PSI* ^+^E9F], but unlike two other prions originating in E9 (Table 1, p<10^−10^). As expected, the prion originating in E9 and transmitted to E9, or that originating in the reference sequence and transmitted to the reference sequence, each maintain their original properties.

Having transferred [*PSI* ^+^ref] to each of the Sup35 polymorphs, we transferred them back to the original host (cured of [*PSI* ^+^]) and re-examined their transmission properties to see if they had changed as a result of their experience (Table 4). The original [*PSI* ^+^ref] transmitted poorly to either Δ19 or E9 hosts, but the ‘experienced’ prions all transmitted better to E9 than the original, indicating selection of a ‘mutant’ prion (Table 4, p<.002, 10^−6^, 10^−10^). Moreover, the prion that passed through Δ19 could transmit 91% to another Δ19 (Table 3), but when passed back to the reference sequence, only transmitted 20% to Δ19 (Table 4). Similarly, the prion passed through E9, and able to transmit to another E9 host at 92% (Table 3), once passed back to the reference host could only transmit 46% to E9 (Table 4).

**Table 4:** Does passage through a Sup35 polymorph change [*PSI* ^+^] transmission properties?

Donor	Recipient allele	Ade+ cytoductant	Total cytoductants	% Ade+
[*PSI^+^* 779-6A]Δ19/779-6A	Reference	188	188	100
	Δ19	38	194	20
	E9	112	175	64
[*PSI^+^* 779-6A]E9/779-6A	Reference	130	130	100
	Δ19	6	117	5
	E9	69	149	46
[*PSI^+^*779-6A]Ref/779-6A	Reference	176	177	99
	Δ19	17	175	10
	E9	55	167	33

Cytoductions of the form ref→polymorph→ref→polymorph were carried out (where ref is strain 799-6A or the same cured of [*PSI* ^+^]). One cytoductant of each ref→polymorph was cytoduced to ref, and five of those cytoductants were each used as donors to each of the three polymorphs. Summed data is shown; the complete data set is shown in Table S6.

These results indicate that the predominant variant has changed. But is this change due to mistemplating as the prion passes from Sup35 molecules with one sequence to those with a different sequence, or is there an ensemble of variants present within the population that can be selected based on the specific selection pressure, to be visible with a specific transmission phenotype?

### Dynamic cloud of prion variants

If the population contains an array of prion variants from which one or another can be selected, one might expect these to segregate during mitotic growth, much as differently marked plasmids sharing the same replicon or mitochondrial genomes will segregate mitotically, even without exposure to a selective condition. In contrast, if the changes in prion variant are due solely to mistemplating when a prion crosses a transmission barrier to a different sequence, then the transmission pattern should not change substantially even after extensive propagation in the original strain. We designed this experiment to separate the mitotic segregation phase, in which there was no change of Sup35p polymorph, from the transmission phase, in which the test of prion variant is then made by cytoduction to the three Sup35 polymorphs.

We subcloned single colonies of the 779-6A [*PSI* ^+^ref] yeast strain (reference Sup35p) without selection on ½ YPD plates for at least 75 generations. Table 5 illustrates our surprising result, that many subclones had transmission profiles considerably different from the parent strain 779-6A. This indicates that there is an ensemble of variants or a prion cloud that has different transmission profiles. We have classified these variants as being type A if they transmit well into reference sequence but poorly into Δ19 and E9 sequences. Type B transmits well into reference and E9 sequences, but poorly into the Δ19 sequence. Type C transmits well into Δ19 and reference sequence, but poorly into the E9 sequence and type D transmits well into all sequences. From this subcloning we now had yeast strains that were carrying prion variants of type B (Y1), type C (Y2) and of type D (Y5). These strains repeatedly display these propagation patterns even after many months in frozen stocks. We then wanted to determine if we had now isolated single variants within the original ensemble so each of three clones, of transmission types B, C and D, were subcloned an additional 75 generations on ½ YPD plates with ten clones of each tested as before. To our surprise these extensively grown subclones of each of the three types still produced clones with an ensemble of prion variants (Table 6). Even the Y1 strain, which did not initially propagate into the Δ19 sequence, produced subclones with a variety of transmission profiles.

**Table 5:** Subclones of [*PSI* ^+^ref] develop divergent transmission properties without selection.

Donor	Recipient allele	Ade+ cytoductant	Total cytoductants	% Ade+	p value^a^	Transmission type
779-6A	Reference	118	120	98		
	Δ19	13	122	11		A
	E9	19	111	17		
Y7	Reference	36	38	95	>.2	
	Δ19	2	40	5	>.3	B
	E9	17	35	**49**	<10^−4^	
Y5	Reference	46	50	92		
	Δ19	13	33	**39**	<10^−4^	D
	E9	20	46	**43**	<10^−3^	
Y1	Reference	86	90	96		
	Δ19	0	51	0		B
	E9	74	101	**73**	<10^−10^	
Y2	Reference	52	55	95		
	Δ19	30	50	**60**	<10^−10^	C
	E9	4	52	8		
Y3	Reference	31	38	82		
	Δ19	16	45	**36**	<10^−4^	D
	E9	14	36	**39**	.006	
Y4	Reference	30	32	94		
	Δ19	0	32	0		B
	E9	10	37	**27**	.02	
Y6	Reference	35	39	90		
	Δ19	23	48	**48**	<10^−7^	D
	E9	11	34	**32**	.02	
Y9	Reference	53	53	100		
	Δ19	19	41	**46**	<10^−6^	C
	E9	8	52	15		
Y10	Reference	67	67	100		
	Δ19	4	37	11		B
	E9	19	39	**49**	<10^−4^	
Y11	Reference	41	42	98		
	Δ19	1	35	3		A
	E9	2	32	6		
Y8	Reference	58	61	95		
	Δ19	4	43	9		A
	E9	4	35	11		
Y12	Reference	42	51	82		
	Δ19	9	49	18		A
	E9	5	38	13		

Twelve subclones of 779-6A were grown for >75 generations and single clones were then amplified and used as cytoduction donors to the three polymorphs. **Bold** figures are transmissions between polymorphs that are more efficient than when the donor was the parent strain 779-6A (top three lines). The p values shown are the probability that the results observed would be obtained by chance if there were in fact no difference between the indicated cytoduction from the subclone and the corresponding cytoduction from the parent strain. The p values are calculated as described in Methods and indicate the probability that the difference between the indicated result with Yx as donor and that with the parent strain 779-6a as donor is due to chance. Transmission types are listed in the text.

**Table 6:** Instability of transmission variants on extensive mitotic growth.

Donor	Recipient allele	Ade+ cytoductant	Total cytoductants	% Ade+		Transmission type
Y5	Reference	46	50	92		
	Δ19	13	33	**39**		D
	E9	20	46	**43**		
Y1	Reference	86	90	96		
	Δ19	0	51	0		B
	E9	74	101	**73**		
Y2	Reference	52	55	95		
	Δ19	30	50	**60**		C
	E9	4	52	8		
Y1-1	Reference	32	32	100		
	Δ19	4	25	16		B
	E9	16	25	64		
Y1-2	Reference	16	16	100		
	Δ19	10	30	**33**	10^−4^	D
	E9	7	16	44		
Y1-3	Reference	30	30	100		
	Δ19	0	34	0		B
	E9	37	50	74		
Y1-4	Reference	35	35	100		
	Δ19	15	42	**36**	<10^−5^	D
	E9	14	40	35		
Y1-5	Reference	48	48	100		
	Δ19	2	23	9		A
	E9	8	35	**23**	<10^−5^	
Y1-6	Reference	25	25	100		
	Δ19	0	10	0		A
	E9	5	25	**20**	<10^−5^	
Y1-7	Reference	56	57	98		
	Δ19	0	41	0		B
	E9	21	36	58		
Y1-8	Reference	49	50	98		
	Δ19	0	36	0		B
	E9	30	40	75		
Y1-9	Reference	40	40	100		
	Δ19	7	36	19		A
	E9	9	35	**26**	<10^−6^	
Y1-10	Reference	44	45	98		
	Δ19	0	29	0		B
	E9	20	42	48		
Y2-1	Reference	30	31	97		
	Δ19	6	38	**16**	<10^−4^	B
	E9	18	40	**45**	<10^−4^	
Y2-2	Reference	48	50	96		
	Δ19	24	70	34		C
	E9	3	30	10		
Y2-3	Reference	36	37	97		
	Δ19	8	45	**18**	<10^−4^	B
	E9	31	54	57		
Y2-4	Reference	33	33	100		
	Δ19	18	34	53		D
	E9	14	32	**44**	<10^−4^	
Y2-5	Reference	24	24	100		
	Δ19	8	30	27		D
	E9	12	37	**32**	0.0015	
Y2-6	Reference	52	58	90		
	Δ19	15	44	34		D
	E9	13	35	**37**	<10^−3^	
Y2-7	Reference	41	42	98		
	Δ19	6	35	**17**	<10^−4^	A
	E9	8	42	19		
Y2-8	Reference	41	48	85		
	Δ19	18	30	60		C
	E9	8	45	18		
Y2-9	Reference	41	42	98		
	Δ19	16	32	50		C
	E9	7	41	17		
Y2-10	Reference	49	49	100		
	Δ19	16	55	29		C
	E9	10	38	26		
Y5-1	Reference	35	37	95		
	Δ19	1	19	5	<0.01	A
	E9	3	17	18	<0.05	
Y5-2	Reference	22	22	100		
	Δ19	0	13	0	<0.01	B
	E9	11	16	69		
Y5-3	Reference	40	45	89		
	Δ19	15	30	50		D
	E9	10	32	31		
Y5-4	Reference	30	30	100		
	Δ19	7	21	33		D
	E9	21	33	64		
Y5-5	Reference	17	17	100		
	Δ19	12	32	38		D
	E9	11	35	31		
Y5-6	Reference	32	32	100		
	Δ19	10	27	37		D
	E9	23	27	85		
Y5-7	Reference	20	20	100		
	Δ19	7	15	47		D
	E9	10	23	43		
Y5-8	Reference	11	22	50		
	Δ19	4	20	20		D
	E9	10	20	50		
Y5-9	Reference	27	30	90		
	Δ19	35	40	88		D
	E9	35	40	88		
Y5-10	Reference	22	25	88		
	Δ19	0	9	0	0.01	B
	E9	9	23	39		

From each of subclones Y1, Y2 and Y5 from Table 5 were isolated ten subclones, which were then propagated a further >75 generations and clones were amplified and used as cytoduction donors to the three polymorphs. The results from Table 5 for Y1, Y2 and Y5 are reproduced at the top for comparison. The p values are calculated as described in Methods and indicate the probability that the difference between the indicated result with Yx-y as donor and that with the parent strain Yx as donor is due to chance. Transmission types are listed in the text.

To determine if the appearance of different predominant variants was due to some unrecognized selective pressure on these strains while propagating on ½ YPD plates, the subcloning was performed in liquid YPD media maintaining the culture in exponential growth phase throughout. Once cell density reached 0.3 absorbance units at 600 nm the cultures were diluted, transferring only 1000 cells to a fresh culture, a process continued for at least 84 generations. Even under exponential growth phase (Table S2), an array of transmission profiles was observed similar to that in Table 6.

The presence of changed transmission patterns in a majority of the clones without any selection having been applied made it clear that the changes were not due to a chromosomal mutation. Nonetheless, we tested for such a chromosomal change by curing [*PSI^+^*] from Y5 by growth on guanidine, and cytoducing cytoplasm from Y1, Y2 or Y5 into strain 4830 and then 8 cytoductants from each were cytoduced into a rho° derivative of the cured Y5 (Table S3). These cytoductants were then cytoduced into recipients each carrying one of the three *SUP35* polymorphs. In each case the transmission pattern followed that of the original Y1, Y2, or Y5 donor of cytoplasm, rather than the Y5 pattern of the recipient (Table S3), confirming that the change was due to a new variant of [*PSI^+^*] and not a chromosomal change. The frequency with which the transmission pattern changed without selection or protein over expression is orders of magnitude higher than for the generation of any new prion, and the fact that the change is one of changing the specificity of transmission to different Sup35p polymorphs proves that it is indeed a change of [*PSI^+^*], and not the generation of some other prion.

To further test the presence of an ensemble of prion variants, one subclone of Y1, which had the same profile as the parent, not being able to transmit into the Δ19 sequence, was subcloned for an additional 75 generations. As shown in Table S4, subclones were obtained with various profiles some with very good transmission into the Δ19 sequence containing strain. These results indicate that a single variant had not been selected and that an ensemble or cloud of prion variants must exist with a dynamic propagation pattern under non-selective conditions. Each isolate has a specific transmission pattern, even after frozen storage for many months (Table S5). We infer that during growth, events must allow for a stochastic shift of the ensemble to allow for isolation of variants with specific reproducible transmission patterns.

### Wild [*PSI* ^+^] transmission

[*PSI* ^+^] is rare in wild strains [33], but was found in 9 of 690 wild isolates [49], each expressing the reference Sup35 (ref. [49] and Amy Kelly, personal communication). How do these wild [*PSI* ^+^] variants respond to the intraspecies barriers we previously reported [20]? We used both reference sequence and E9 sequence Sup35 fused to GFP and could see dots in the reported wild [*PSI* ^+^] strains 5672, UCD#885, UCD#978 and UCD#2534, though infrequently, but not in strains UCD#521, 587, 779, 824, 939 (Figure S1). To test these strains genetically for nonsense suppression, we crossed the wild strains with strain 4972 (Table S1), carrying the [*PSI* ^+^]-suppressible *ade1-14* marker, and tested dissected tetrads to determine if *ade1-14* is suppressed. We found that for seven of the wild strains, *ade1-14* was weakly suppressed in the segregants, and this suppression could be cured by growth in the presence of guanidine, which is known to cure the [*PSI* ^+^] prion. We could not obtain tetrads from diploids formed with strain UCD# 978 and strain 5672 gave poor spore germination.

**Figure 1:**
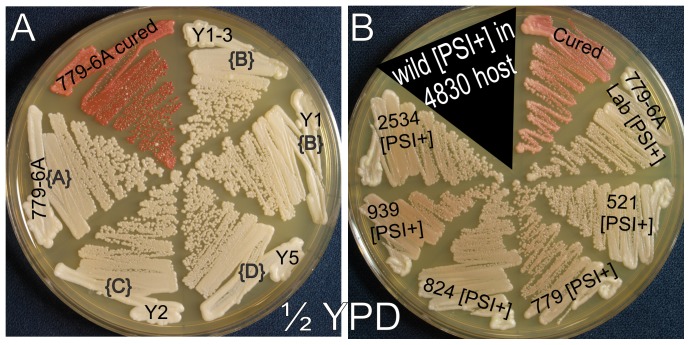
[*PSI* ^+^] variants with distinct transmission properties can have identical “strong” or “weak” phenotypes. A. [*PSI* ^+^] strains derived from 779-6A by extensive non-selective subcloning have different transmission patterns, but identical “strong” phenotypes. B. [*PSI* ^+^] prions in wild *S. cerevisiae* isolates were moved into strain 4830 for direct comparison of prion intensity. Each is “weak”, although transmission to Sup35p polymorphs varies as indicated. [A], [B], [C] and [D] refer to the transmission types shown in Table 5.

The transmission of the wild [*PSI* ^+^] isolates into cells expressing the Sup35 polymorphs in strains 4828 and 4830 by cytoduction is shown in Table 7. The wild [*PSI* ^+^] strains transmit well into the reference sequence, but most showed poor transmission to one or both of the Δ19 or E9 sequences (Table 7). All four transmission patterns were observed (Table 7), but all of the isolates were ‘weak’ [*PSI* ^+^] (Figure 1B). Thus, each of the strains tested transmitted [*PSI* ^+^] even though several did not show dots with Sup35NM-GFP. Of course, their presumed independent origin means that these wild isolates are not derived from one prion cloud.

**Table 7:** Wild [*PSI* ^+^] prion isolates are largely sensitive to polymorph-determined transmission barriers.

[*PSI^+^*] Source	Donor	Recipient 4830	Ade+ cytoductant	Total cytoductants	% Ade+	Transmission type
Laboratory	779-6a	Reference	45	48	94	
		Δ19	5	50	10	A
		E9	4	40	10	
Wild strain	DB01-8C	Reference	28	36	78	
UCD521		Δ19	8	47	17	A
		E9	13	60	22	
Wild strain	DB03-12A	Reference	27	30	90	
UCD779		Δ19	0	25	0	A
		E9	1	35	3	
Wild strain	DB04-3B	Reference	59	63	**94**	
UCD824		Δ19	8	50	16	B
		E9	67	82	**82**	
Wild strain	DB06-5B	Reference	42	50	84	
UCD939		Δ19	12	55	22	A
		E9	9	47	19	
Wild strain	DB07-7C	Reference	48	53	91	
UCD2534		Δ19	40	53	75	D
		E9	50	70	71	
		**Recipient 4828**				
Wild strain	DB02-1D	Reference	43	65	66	
UCD587		Δ19	132	132	100	C
		E9	14	60	23	
Wild strain	DB05-7C	Reference	82	82	100	
UCD885		Δ19	28	62	45	D
		E9	65	87	75	
Wild strain	DB07-3B	Reference	112	112	100	
UCD2534		Δ19	96	96	100	D
		E9	91	91	100	

Spores of wild *S. cerevisiae* reported to be [*PSI* ^+^] [49] were crossed with strain 4972 and meiotic segregants showing weak, guanidine-curable suppression of *ade1-14* were used as cytoduction donors.

### Strong [*PSI* ^+^] includes several prion variants

Variants of [*PSI* ^+^] may be weak or strong in phenotype, stable or unstable in propagation, and have various responses to deficiency or over expression of chaperones or other cellular components, have different patterns of ability to cross species barriers, and, as shown here, to cross intraspecies transmission barriers. To what extent these various parameters are correlated is largely unknown. We tested the several prion variants derived from the [*PSI* ^+^] in strain 779-6A with different transmission patterns for their ‘strong’ vs ‘weak’ character (Figure 1A). We note that, with identical chromosomal genotype, they are indistinguishable in the ‘strength’ parameter in spite of having substantially different transmission properties. As noted above, the wild [*PSI* ^+^] variants are indistinguishably ‘weak’, but have different transmission patterns to the Sup35 polymorphs.

## Discussion

Yeast prion variants are distinguishable based on intensity of the prion phenotype, stability or instability of prion propagation, sensitivity of prion stability to overproduction or deficiency of several chaperones and other cellular components and ability to overcome barriers to transmission between species [30], [31], [37]–[41] – or even within species, the last documented here for transmission across the barriers found in wild strains of *S. cerevisiae*. Yeast prion amyloids are all folded parallel in-register β-sheet structures [21], [50], [51], but within this architectural restraint, different prion variant structures are proposed to vary in the extent of the β-sheet structure (how much of the N and M domains are in β-sheet), the locations of the folds in the sheets and the association of protofilaments to form fibers.

We find that separation of prion variants based on sensitivity to intra-species barriers cuts across separation based on ‘strong’ vs ‘weak’ assessment of strength of prion phenotype. The four transmission variant types derived from the [*PSI* ^+^] in strain 779-6A were all strong [*PSI* ^+^], like the parent prion. Interestingly, the prions in wild strains were all weak [*PSI* ^+^], presumed to arise independently and thus not part of the same ‘prion cloud’, but fell into the same four transmission variant types. Likewise, two similarly ‘weak’ [*PSI* ^+^] variants showed different transmission across a barrier set up by deletions in the prion domain [52]. These results show that prion variant uniformity is not demonstrated by showing uniformity of a single property (for example, colony color). It is unlikely that the variation in transmission barriers observed are due to a prion other than [*PSI^+^*] because the sequences of Sup35p are involved, and no yeast prion is known to arise at a frequency high enough to explain our results.

After crossing an intraspecies barrier, we find that the [*PSI* ^+^ref] examined is unstable in its new host, emphasizing the effectiveness of these barriers. We also find that the rare [*PSI* ^+^] prions found in wild strains are, in most cases, sensitive to the intraspecies barriers, suggesting that these barriers have evolved to protect yeast from the detrimental effects of this prion.

The [*PSI* ^+^] in strain 779-6A, with the reference Sup35p sequence, showed a reproducible strong preference for the reference sequence, transferring only very inefficiently to the Δ19 or E9 Sup35 backgrounds. However, simple mitotic growth of this strain resulted in the mitotic segregation of at least four variants distinguished by their abilities to cross intraspecies barriers. These variants were stable and reproducible with limited expansion of the corresponding clones, but following many generations of growth, each of those tested gave rise again to the same four general classes of subclones. Prion mutation is well documented in mammals and in yeast under selective conditions [30], [46], [47], [53], and Weissmann's group has suggested that prions resistant to a drug can arise during prion propagation in tissue culture cells in the absence of the drug [54], [57]. We observe changes in the predominant prion variant under non-selective conditions in vivo. Selection only happens during the test, when cytoplasm is passed by cytoduction from the subclones to be tested to the recipient expressing one of the three Sup35p polymorphs. A new prion variant, recently described by Sharma and Liebman [55], may represent a phenomenon similar to that described here. Certain induced [*PSI^+^*] clones continually gave off subclones that were a mixture of strong and weak variants, what the authors called “unspecified [*PSI^+^*]”.

Although multiple de novo prion generation events in forming amyloid in vitro result in multiple prion variants on transfection into yeast, even a [*PIN* ^+^] cell generates [*PSI* ^+^] clones too rarely to explain our results as de novo prion generation. Rather, mis-templating must be the mechanism of generation of variant diversity that we are observing. Our results imply that there must be a finite rate of amyloid mis-templating that is not due to a mismatch of two prion protein sequences. In spite of extensive purification by mitotic growth and subcloning, we were unable to obtain a prion variant that was completely stable in its transmission pattern to polymorphs. These results are consistent with the ‘prion cloud’ hypothesis [56], [57], in which it is supposed that even a prion variant purified by end-point titration consists of a major variant as well as an array of minor variants. This production of new prion variants during non-selective growth is analogous to the generation of RNA virus mutants during viral replication (reviewed in ref. [58]), in which a cloud of sequence variants accumulate because of the error-prone nature of RNA-dependent RNA polymerases.

The segregation of a mixed prion population could be considered analogous to the segregation of differently marked plasmids with the same replicon. The latter situation has been carefully examined by Novick and Hoppenstadt [59], who find that the fraction of cells remaining with a mixture of plasmids is H = H_0_ [(N−1)(2N+1)/(2N−1)(N+1)]^n^ , where H_0_ is the starting fraction of mixed cells, N is the copy number of the plasmid, and n is the number of generations [59]. Random replication of plasmids and equal partition at mitosis is assumed. One result of this treatment is that after N generations, H≈0.36 H_0_.

The copy number in the case of yeast prions might be taken as the ‘seed number’ determined by the methods developed by Cox et al. [60], found to be ∼20–120 for the strains examined. The assumption of equipartition is probably not accurate here, since yeast daughter cells are smaller than mother cells [60]. Moreover, the sticky nature of amyloids might suggest that progeny filaments might stick to parent filaments exaggerating this effect. We have propagated our [*PSI* ^+^] strains for a number of generations comparable to the presumed copy number, so segregation of different prions is not surprising.

**Figure 2:**
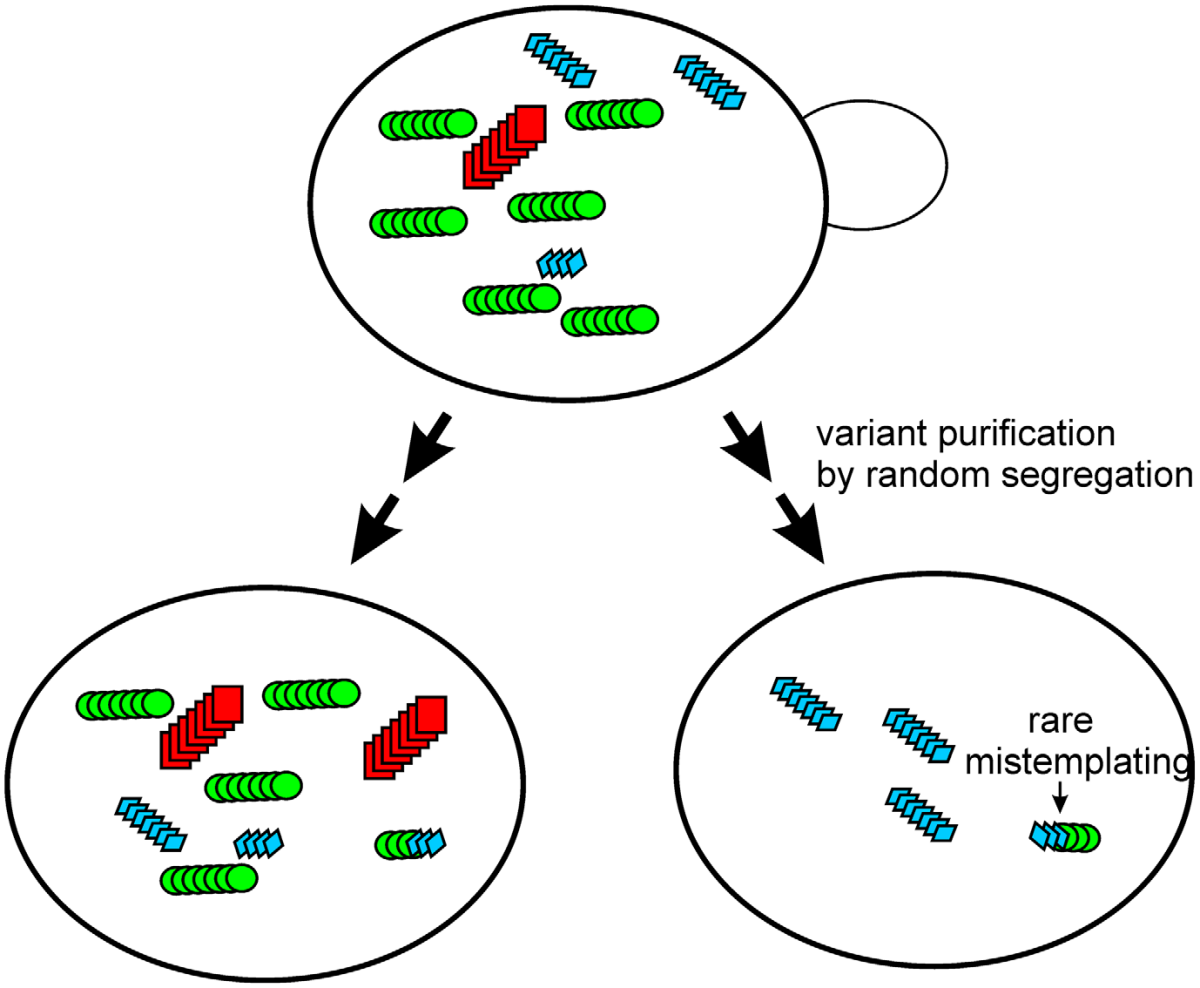
The prion cloud model **[56]**, **[57]** applied to yeast. Segregation of different prion variants on mitotic growth is followed by re-emergence of different variants, presumably due to mis-templating.

However, we find that even when we have apparently purified a variant, further non-selective growth and subcloning leads to further appearance of the full range of variants among the progeny (Figure 2). This indicates that we are not only observing segregation, but also the (repeated) generation of variants during growth. While varying with respect to transmission, they remain ‘strong’ variants, suggesting that the structural differences responsible for this transmission barrier differ from those involved in the strong vs. weak differences. King has shown that residues 1–61 are sufficient to propagate strong vs weak prion strains [8], [61], but the sequence differences among the Sup35 polymorphs are outside this area, and transmission variants may thus largely differ in the region C-terminal to the 1–61 area, perhaps a region with more variable structure. Other studies have indicated effects of this region on propagation of some prion variants [52], and β-sheet structure of Sup35NM amyloid extends throughout N and even into M [21], [22].

## Materials and Methods

### Nomenclature

We refer to the standard laboratory yeast sequence [62]–[64] as the ‘reference sequence’. Two common sequence polymorphs found within the wild population were used. The first, with deletion of 19 amino acids from residues 66 to 84 and the G162D change, is referred to as Δ19, and the other includes N109S, G162D, D169E, P186A, T206K, H225D and is denoted E9 [20]. A prion originating with the Sup35p sequence of strain E9, for example, but being propagated in a strain expressing only the reference sequence will be designated [*PSI* ^+^E9]ref, in analogy with similar nomenclature for [URE3] [31]. Cytoductants (see below) generated with strain A as donor and strain B as recipient are denoted A→B. They have the nuclear genotype of strain B and the cytoplasmic genotype of both A and B. In an abuse of language, we often use “[*PSI* ^+^E9]ref was transferred to Sup35 Δ19” to mean “[*PSI* ^+^E9]ref was transferred to cells expressing Sup35 Δ19”.

### Scoring the [*PSI* ^+^] prion

Sup35p is a subunit of the translation termination complex, and the incorporation of a large proportion of Sup35p into the prion amyloid filaments makes it inactive, resulting in increased read-through of termination codons. This is measured by read-through of *ade2-1*, with an ochre termination codon in the middle of the *ADE2* gene. In addition to *ade2-1*, strains carry the *SUQ5* weak suppressor mutation, which leaves cells Ade- unless the [*PSI* ^+^] prion is also present [2].

### Strains, plasmids, and media

The strains used are listed in Table S1. Plasmids used containing reference, Δ19 or E9 sequences were generated as described [20]. All yeast media and plates contained 20 µM copper sulfate unless noted. Rich and minimal media (YPAD and SD) are as described [65]. Only nutrients required by the strains used in a given experiment were added to minimal plates.

### Cytoduction

Cytoplasm may be transferred from one strain to another utilizing the *kar1-1* mutation [66], defective for nuclear fusion. Cells fuse, but the nuclei do not fuse, and nuclei separate at the next cell division. However, cytoplasmic mixing has occurred, and so a genetic element (prion or mitochondrial DNA) present in one strain (identified by its nuclear genotype) will be transferred to the other. We use transfer of mitochondrial DNA as a marker of cytoplasmic transfer, and score prion transfer. Reference, Δ19 or E9 sequence plasmids were transformed into both laboratory strains 4828 and 4830, loss of p1215 (*URA3 SUP35C*) was selected by growth on 5-fluoroorotic acid media and Ade- transformants were made rho° by growth on YPAD containing 1 mg/ml ethidium bromide. Donor and recipient strains at high density were mixed in water at a ratio of about 5∶1, and the mixture was spotted onto a YPAD plate. After 18 hours at room temperature, the mating mix was streaked for single colonies on media selective against growth of the donor strain. Clones are shown to be cytoductants by their growth on glycerol and failure to grow on media selective for diploids. As further tests of a sample confirm, Ade+ cytoductants are judged to have received and propagated [*PSI* ^+^].

### Subcloning

[*PSI* ^+^] Strain 779-6A [48] was streaked to single colonies on ½ YPD media and twelve colonies were selected, named Y1-Y12. These isolates were streaked to single colonies three additional times, each time selecting just one colony for further propagation. From the third plate a single colony was selected and expanded on ½ YPD, and cells from this plate were used for cytoduction. From dilution tests there are approximately 2×10^7^ cells per colony, indicating a total of at least 75 generations of growth of clones Y1-Y12 before cytoduction. Additional subclones were handled in the same manner with only ten colonies selected from the initial ½ YPD plate. In experiments to rule out selection during stationary growth phase, subclones of Y1 and Y2 were grown in a 125 ml Erlenmyer flask containing 25 ml of liquid YPD medium. When A_600_ reached 0.3, the culture was diluted, transferring 1000 cells of each to a fresh flask. These subclones were propagated in exponential phase for 84 generations and were then streaked for single colonies on ½ YPD plates. After one day of growth on ½ YPD, 10 subclones were selected for each of Y1 and Y2, expanded and tested for transmission via cytoduction.

### Wild [*PSI* ^+^] strains

Strains reported to be [*PSI* ^+^] [49] were obtained from the UC Davis Department of Viticulture and Enology culture collection. The cultures were first tested to determine if dots were visible using either reference sequence Sup35NM-GFP pDB65 or E9 sequence Sup35NM-GFP pDB81 [20]. Images were obtained with a Nikon Eclipse TE2000-U spinning disc confocal microscope with 100× NA 1.4 Nikon oil lens with 1.5× magnifier and captured with a Hamamatsu EM-CCD ImagEM digital camera with IPLab version 4.08. Wild strains were sporulated and spores were crossed on rich medium with strain 4972 selecting G418-resistant prototrophs. The diploids formed were again sporulated and tetrads were dissected for each wild strain except for strain 978, whose diploid with 4972 would not sporulate. Ade positive segregants were tested for guanidine curing using two successive streaks on YPAD with 5 mM guanidine. *MATα* strains were cytoduced into strain 4830 carrying pRS316 (*URA3*) for selection. *Lys2* mutants of *MATa* strains were selected on plates with DL-α-aminoadipic acid as a nitrogen source [67]. Selected strains were retested for Ade positive growth and curing and cytoduced into strain 4828.

### Statistical methods

The cytoduction data follows the binomial distribution, because each data point expresses two alternative results, transmission of [*PSI^+^*] or failure of its transmission. However, because of the large number of observations, the results should be approximately normally distributed. We want to calculate the probability that two sets of data are due to chance. Let p_1_ and p_2_ be the observed proportions of transmission in cytoductions 1 and 2, and n_i_ the number of cytoductants tested in each experiment. Let p = (p_1_n_1_+p_2_n_2_)/(n_1_+n_2_) be the average of the proportion of transmission in the two experiments. The estimated standard error of the difference between the two proportions is
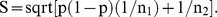
The null hypothesis is that cytoductions 1 and 2 are samples from the same population with transmission efficiency p and standard error S. Then the expected proportions are expected to be the same and their difference is expected to be zero. [(p_1_−p_2_)−0]/S = z = the number of standard deviations that the observed difference in proportions differs from the expected difference (0). The frequency of “z” being greater or equal to the observed value (assuming the null hypothesis) is obtained from a table of the normal distribution. The calculated “p values” are shown in the tables and at appropriate points in the text.

Cytoductants examined have been treated as independent since the chance that they represent sister cells is close to zero. This is because cytoductant mixtures were incubated at 20C where the cells divide slowly and because only about 30 cells were examined from several million in the zygote mixture on each plate.

## Supporting Information

Figure S1 Aggregation of Sup35-GFP in reported wild [*PSI* ^+^] strains. Wild strains reported to carry [*PSI* ^+^] [49] were transformed with plasmids expressing Sup35NM(ref)-GFP or Sup35(E9)-GFP and carrying *kanMX*, and examined microscopically as described in Methods. Strains UCD#521, 779 and 824 do not show obvious dots. Strains UCD#885, 978 and 2534 show dots which appear smaller than in the laboratory [*PSI* ^+^] strain 779-6A. Dots in strain 5672 are comparable to those in the laboratory strain. Strains UCD#587 and 939 were indeterminate.
(TIF)

Table S1 Strains of *Saccharomyces cerevisiae*.
(DOC)

Table S2 Transmission by subclones isolated after 84 generations of exponential growth in liquid media. Transmission by parent strains (Y1, Y2, Y5) is shown at the top for comparison. After growth for ∼84 generations in liquid medium as described in Methods, single colonies were isolated, grown and used as cytoduction donors to cells expressing the three Sup35 polymorphs.
(XLS)

Table S3 Y1, Y2 and Y5 transmission phenotypes are not due to chromosomal mutations. Strain Y5 was cured of the [*PSI^+^*] prion using growth in the presence of guanidine, and of the mitochondrial DNA using ethidium bromide. [*PSI^+^*] was cytoduced from strains Y1, Y2 and Y5 to strain 4830 rho°, and ten cytoductants from each were then used as cytoduction donors to the [psi−] rho° derivative of Y5. One cytoductant from each of these cytoductions was picked and used as a donor into recipients expressing each of the three Sup35 polymorphs.
(XLS)

Table S4 Y1 Subclones of Y1-3 diverge in transmission phenotype indicating inability to purify a specific variant of PSI transmission.
(XLS)

Table S5 Y1 transmission is unchanged after months in frozen stock.
(XLS)

Table S6 Cytoductions of individual clones summed in Table 4.
(XLS)
