# Correction: The [*PSI*^+^] Prion Exists as a Dynamic Cloud of Variants

**DOI:** 10.1371/journal.pgen.1006591

**Published:** 2017-02-03

**Authors:** The PLOS Genetics Staff

In the Materials and Methods section, there is an error in the equation under the subheading “Statistical methods”. The publisher apologizes for the error. Please view the complete, correct equation here:

$$\text{S }=\text{ sqrt}\left[\text{p}\left(\text{1}-\text{p}\right)\text{/}\left({\text{n}}_{1}+{\text{n}}_{2}\right)\right]$$
