# Supplementary figures and images for: The [PSI +] Prion Exists as a Dynamic Cloud of Variants
Source: PLoS Genet. 2013 Jan 31;9(1):e1003257. doi: 10.1371/journal.pgen.1003257 (PMC3561065; doi:10.1371/journal.pgen.1003257)

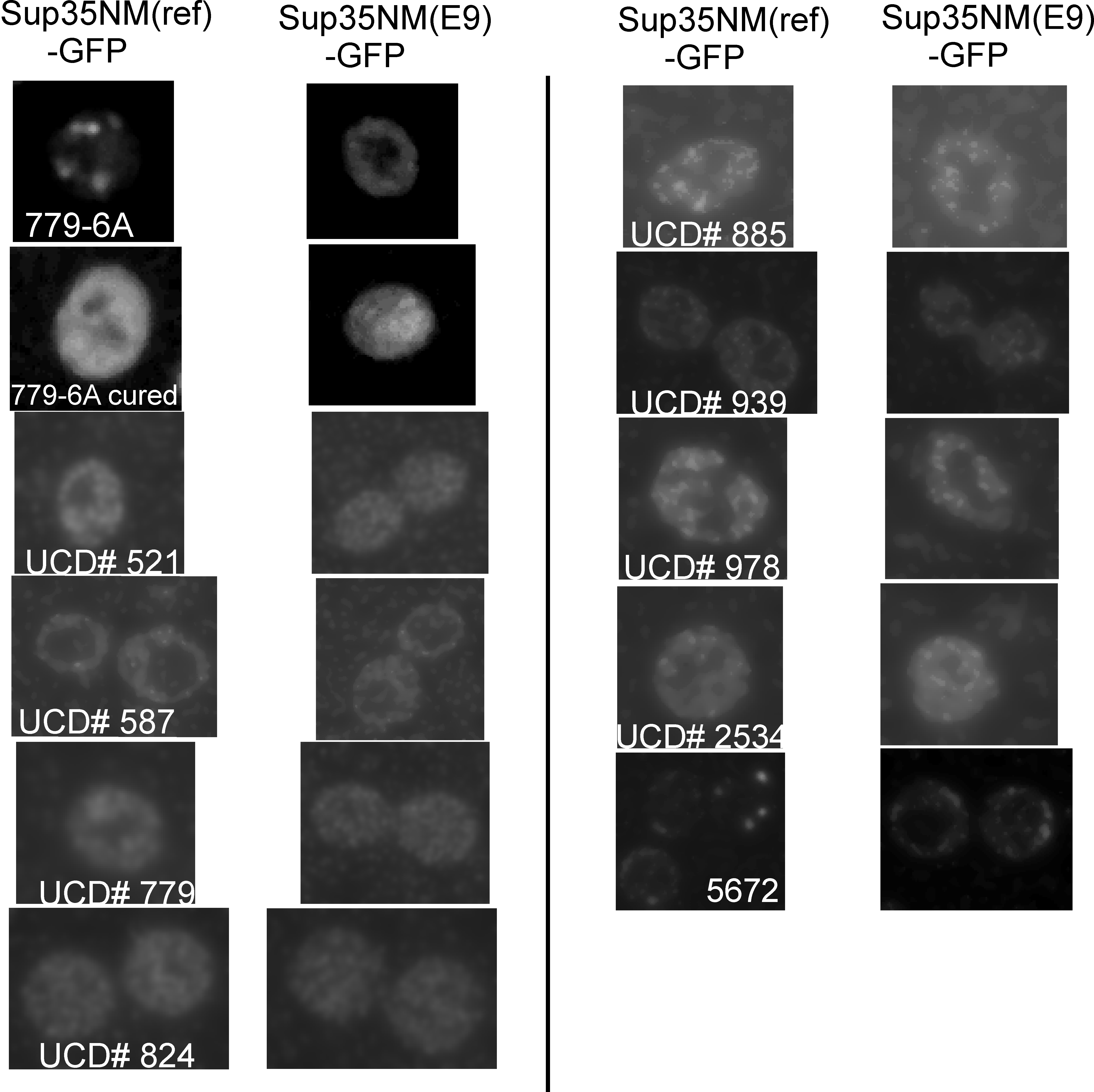

Supplement: Figure S1 — Aggregation of Sup35-GFP in reported wild [PSI +] strains. Wild strains reported to carry [PSI +] [49] were transformed with plasmids expressing Sup35NM(ref)-GFP or Sup35(E9)-GFP and carrying kanMX, and examined microscopically as described in Methods. Strains UCD#521, 779 and 824 do not show obvious dots. Strains UCD#885, 978 and 2534 show dots which appear smaller than in the laboratory [PSI +] strain 779-6A. Dots in strain 5672 are comparable to those in the laboratory strain. Strains UCD#587 and 939 were indeterminate. (TIF) [file pgen.1003257.s001.tif]
