# Supplementary material for: The [PSI +] Prion Exists as a Dynamic Cloud of Variants
Source: PLoS Genet. 2013 Jan 31;9(1):e1003257. doi: 10.1371/journal.pgen.1003257 (PMC3561065; doi:10.1371/journal.pgen.1003257)
Supplement: Table S1 — Strains of Saccharomyces cerevisiae. (DOC) [file pgen.1003257.s002.doc]

|  | |  | |  | |  | |  | |  |  |  | |  |  |
| --- | --- | --- | --- | --- | --- | --- | --- | --- | --- | --- | --- | --- | --- | --- | --- |
| Supplemental Table S1. Strains of *Saccharomyces cerevisiae.* | | | | | | | | | | |  |  | |  |  |
| Strain Number | | Genotype | |  | |  | |  | |  |  |  | |  |  |
| 4828 | | *MAT*α *ade 2-1 SuQ5 trp1 kar1-1 his3 leu2 ura3 sup35::kanMX* [PIN+] [psi-] p1215: *CEN URA3* *SUP35MC* | | | | | | | | | | | | |  |
| 4830 | | *MAT***a** *ade 2-1 SUQ5 trp1 kar1-1 lys2 leu2 ura3 sup35::kanMX* [pin-] [psi-] p1215: *CEN URA3 SUP35MC* | | | | | | | | | | | | |  |
| 779-6A | | *MAT*α *ade 2-1 SUQ5 trp1 kar1-1 his3 leu2 ura3* [PSI+] (reference 50) | | | | | | | | | |  | |  |  |
| 4972 | | *MAT***a** *ade 1-14 kar1-1 ho::KanMX leu2 ura3 his4* [PIN+] [psi-] | | | | | | | | |  |  | |  |  |
| 167 | | *MAT*α *his3* | |  | |  | |  | |  |  |  | |  |  |
| 168 | | *MAT***a** his3 | |  | |  | |  | |  |  |  | |  |  |
| DB01-8C | | *MATα ade1-14 ura3 ho::KanMX* [PSI+ from wild strain 521] | | | | | | | | |  |  | |  |  |
| DB02-1D | | *MAT***a** *ade1-14 leu2 his4 lys2* [PSI+ from wild strain 587] | | | | | | | |  |  |  | |  |  |
| DB02-3C | | *MAT*α *ade1-14* *his4 ura3 leu2 ho::KanMX* [PSI+ from wild strain 587] | | | | | | | | |  |  | |  |  |
| DB03-12A | | *MATα ade1-14 his4 ura3 ho::KanMX* [PSI+ from wild strain 779] | | | | | | | | |  |  | |  |  |
| DB04-3B | | *MAT*α *ade1-14 his4 ho::KanMX* [PSI+ from wild strain 824] | | | | | | | | |  |  | |  |  |
| DB05-7C | | *MAT***a** *ade1-14 ho::KanMX leu2 ura3 his4 lys2* [PSI+ from wild strain 885] | | | | | | | | | |  | |  |  |
| DB06-5B | | *MAT*α *ade1-14 his4 leu2 ho::KanMX* [PSI+ from wild strain 939] | | | | | | | | |  |  | |  |  |
| DB07-3B | | *MAT***a** *ade1-14 ho::KanMX leu2 his4 ura3 lys2* [PSI+ from wild strain 2534] | | | | | | | | | |  | |  |  |
| DB07-7C | | *MAT*α *ade1-14 his4 ura3 leu2 ho::KanMX* [PSI+ from wild strain 2534] | | | | | | | | | |  | |  |  |
|  | |  | |  | |  | |  | |  |  |  | |  |  |
| Wild strains | |  | |  | |  | |  | |  |  |  | |  |  |
| **UCD#** | | **Strain** | | **Source** | |  | |  | |  |  |  | |  |  |
| 521 | | Marsala | | Marsala wine | | | |  | |  |  |  | |  |  |
| 587 | | FST C-230 | | Must Semillon | | | |  | |  |  |  | |  |  |
| 779 | | KI-V1116, V86 | | Commercial dry wine yeast | | | | | |  |  |  | |  |  |
| 824 | | 288 | | commercial wine yeast | | | |  | |  |  |  | |  |  |
| 885 | | K103 | | unknown | |  | |  | |  |  |  | |  |  |
| 939 | | Ba99 Lambrusco | | grapes, vineyard, Italy | | | |  | |  |  |  | |  |  |
| 978 | | DGI-299 | | Commerical dry wine yeast | | | | | |  |  |  | |  |  |
| 2534 | | GY | | Commerical dry wine yeast | | | | | |  |  |  | |  |  |
|  | | 5672 | | Lindquist laboratory | | | |  | |  |  |  | |  |  |
|  | |  | |  | |  | |  | |  |  |  | |  |  |
|  |  | |  | |  | |  | |  | |  |  |  | |  |
